# Potential Transmission Pathways of *Streptococcus gallolyticus* subsp. *gallolyticus*

**DOI:** 10.1371/journal.pone.0126507

**Published:** 2015-05-15

**Authors:** Jessika Dumke, Dennis Hinse, Tanja Vollmer, Jochen Schulz, Cornelius Knabbe, Jens Dreier

**Affiliations:** 1 Institute for Laboratory and Transfusion Medicine, Ruhr University of Bochum, Bad Oeynhausen, Germany; 2 Institute for Animal Hygiene, Animal Welfare and Farm Animal Behavior, University of Veterinary Medicine Hannover, Foundation, Hannover, Germany; Centers for Disease Control & Prevention, UNITED STATES

## Abstract

*Streptococcus gallolyticus* subsp. *gallolyticus* (*S*. *gallolyticus* subsp. *gallolyticus*), a member of group D streptococci, is an inhabitant of the animal and human gastrointestinal tract. Furthermore, it is a facultative pathogen which causes e.g. endocarditis, septicemia and mastitis. *S*. *gallolyticus* subsp. *gallolyticus* may be transmitted either directly or indirectly between animals and humans. However, the transmission routes are an unsolved issue. In this study, we present systematic analyses of an *S*. *gallolyticus* subsp. *gallolyticus* isolate of an infective endocarditis patient in relation to isolates of his laying hen flock. Isolates from pooled droppings of laying hens, pooled dust samples and human blood culture were characterized by using multilocus sequence typing (MLST) and DNA fingerprinting. MLST revealed the same allelic profile of isolates from the human blood culture and from the droppings of laying hens. In addition, these isolates showed clonal identity regarding a similar DNA fingerprinting pattern. For the first time, we received a hint that transmission of *S*. *gallolyticus* subsp. *gallolyticus* between poultry and humans may occur. This raises the question about the zoonotic potential of isolates from poultry and should be considered in future studies.

## Introduction

*Streptococcus gallolyticus* subsp. *gallolyticus* was formerly known as *Streptococcus bovis* biotype I. However, because of the ability to hydrolyze gallic acid, it was reclassified as *S*. *gallolyticus* subsp. *gallolyticus* in 2003 [1]. The gram-positive bacterium of Lancefield group D streptococci is a commensal of the human gastrointestinal tract and appears in 2.5 to 15.0% of healthy humans [2, 3]. Furthermore, *S*. *gallolyticus* subsp. *gallolyticus* has been identified in several animal species (e.g. bovine, pigeon, chicken, pig) [4–6]. This opportunistic bacterium is also a facultative pathogen and causes several diseases in animals and humans. As an example, *S*. *gallolyticus* subsp. *gallolyticus* was the causative agent in 24% of human streptococcal endocarditis cases [7]. In addition, several studies showed an association between streptococcal endocarditis and colon cancer [3, 8, 9]. Diseases such as septicemia and meningitis can be triggered by *S*. *gallolyticus* subsp. *gallolyticus* in humans as well as in animals [6, 10].

Many aspects concerning the pathomechanism, the transmission routes and the zoonotic potential of *S*. *gallolyticus* subsp. *gallolyticus* remain unexplained [11]. Direct and indirect transmission pathways are described for other zoonotic pathogens. An indirect transmission by contaminated surfaces, dust (e.g. animal feed, excreta) and by inhaling or swallowing bioaerosols (e.g. bacteria) has been shown [12, 13]. A close contact between animals and humans enhances the risk of transmission for bacteria such as *Streptococcus suis* [14, 15]. The consumption of unpasteurized raw dairy products was the source of *S*. *equi* subsp. *zooepidemicus* infections [16–18]. However, the transmission pathways of *S*. *gallolyticus* subsp. *gallolyticus* are not elucidated. Multilocus sequence typing (MLST) can be an appropriate method to gain insights into transmission pathways, population structures and the zoonotic potential of bacteria [19]. Sequencing of seven housekeeping genes of bacterial strains resulted in specific allelic profiles and each profile determined a sequence type (ST). In the past, MLST was successfully established to identify zoonotic organisms. The typing method, for example, was successfully applied for *S*. *suis* and resulted in the identification of an important outbreak strain in provinces in China [15].

The recently established subspecies-specific MLST scheme was used to characterize the epidemiology of *S*. *gallolyticus* subsp. *gallolyticus* [20]. There are 50 STs determined for *S*. *gallolyticus* subsp. *gallolyticus*. On the one hand, some STs are limited to animals (e.g. ST 45 to pigeons and ST 50 to turkeys) or humans (ST 8); on the other hand, there are STs which are shared by both (animals and humans). The STs 3 and 12, for example, can be found in human blood cultures and in bovines (ST 12 isolate from bovine suffering from mastitis) [20]. The occurrence of the same allelic profiles in animals and humans leads to the assumption that *S*. *gallolyticus* subsp. *gallolyticus* may act as a zoonotic organism [20]. This was also suggested by Shibata *et al*. [21]. However, to date there is no evidence of host specificity, geographic-region-related occurrence or a zoonotic potential of *S*. *gallolyticus* subsp. *gallolyticus*. A comparison between *S*. *gallolyticus* subsp. *gallolyticus* isolates from animals and humans which come into close contact could be a useful tool to investigate the possibility of a transmission between the two species. In this study, we compare isolates from a blood culture of an infective endocarditis patient with isolates of his own laying hen flock by typing and DNA fingerprinting. On the basis of these results, the possibility of transmission between hens and the farmer is discussed.

## Material and Methods

### Case description

In February 2013, a 54-year-old man with an artificial heart valve (implanted three years ago) was admitted to the Heart and Diabetes Center North Rhine-Westphalia with fever and infective endocarditis, which was diagnosed according to the Duke criteria [22]. Gram-positive cocci were isolated from the initial blood culture and antibiotic therapy (penicillin G, gentamicin and amoxicillin) was initiated. The bacteria were identified as *S*. *gallolyticus* subsp. *gallolyticus* by matrix-assisted laser desorption/ionization time-of-flight mass spectrometry (MALDI-TOF MS) and sequencing of *sodA* (encoding the manganese-dependent superoxide dismutase), as described previously [23, 24].

A colonoscopy was performed and no malignant changes were identified in the intestinal tract. The screening of the patient’s fecal sample was negative for *S*. *gallolyticus* subsp. *gallolyticus*.

The study was approved by the ethics commission of the Ruhr University of Bochum Faculty of Medicine, located in Bad Oeynhausen. The patient provided his written informed consent to participate in the study.

### Sampling, isolation and identification of *S*. *gallolyticus* subsp. *gallolyticus*

#### Sampled laying hen flock

**Fig 1:**
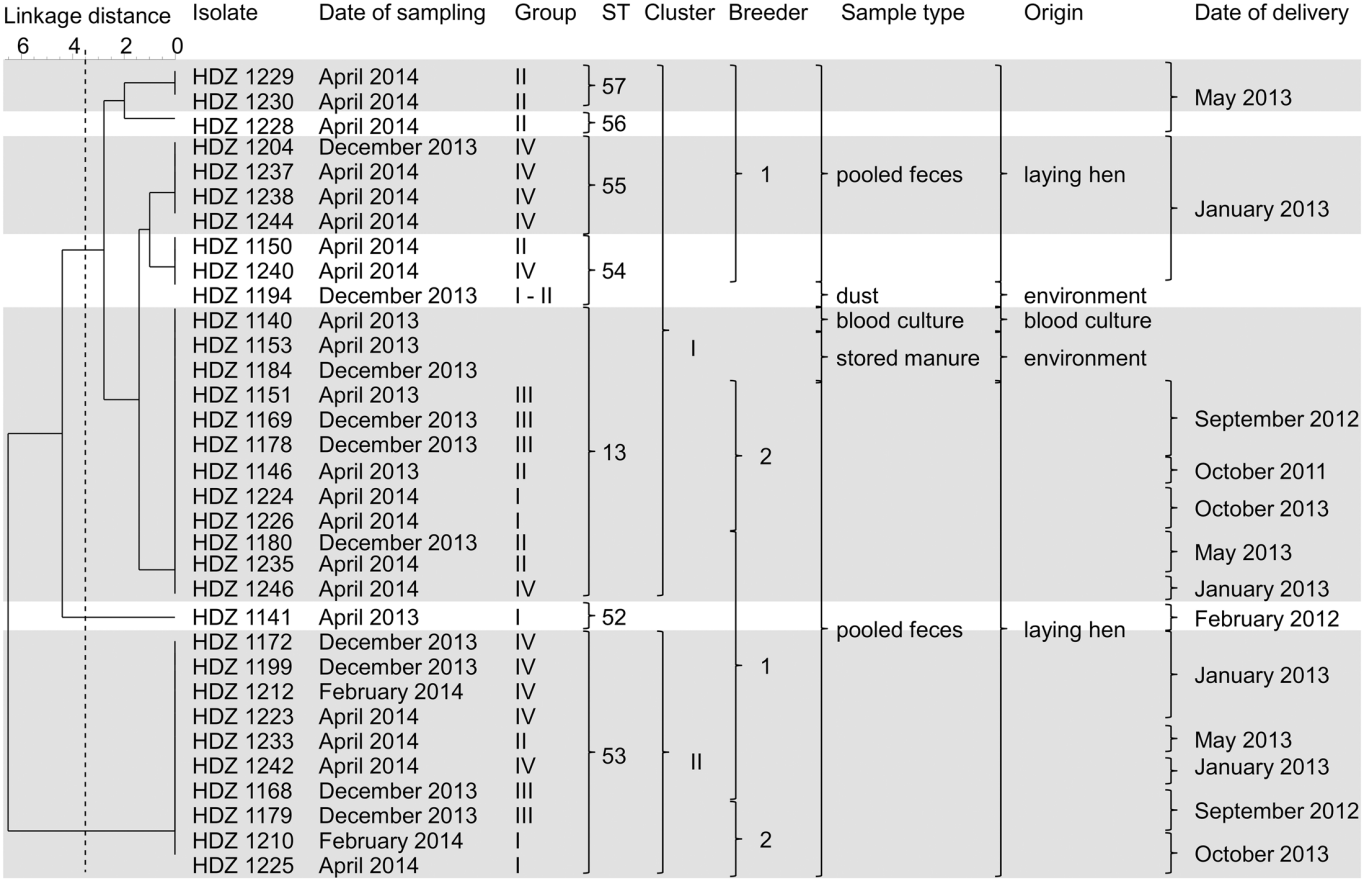
UPGMA dendrogram of 33 *S*. *gallolyticus* subsp. *gallolyticus* isolates. The dendrogram shows bacterial isolates and the corresponding parameters (source of isolate ion, origins of isolation, date of sampling and delivery, sequence types and clusters). The dashed line symbolizes the cluster border, which was calculated based on the SID. The isolates with the same STs are highlighted in grey and white. **Abbreviation: I–IV** isolates from droppings from laying hen group I–IV; **ST** sequence type.

Samples were taken from a multiage small colony-keeping system for laying hens of a German state (North Rhine-Westphalia). The laying hen flock consisted of four groups of 1,200 laying hens (with one exception (1,100 hens): laying hen group I, arrived in February 2012), which originated from two different breeders. Sampling was performed during a twelve-month period from April 2013 to April 2014. Table 1 and Fig 1 give detailed information about the sampling within different laying hen groups and the surrounding of the flock.

**Table 1:** Sampling of a laying hen flock and results of cultivation and characterization of isolates.

Date of sampling	Source	Age of hens	Sample type	Cultiv-ation*	Isolate	ST**
February/2013	human	-	blood culture	+^(#)^	HDZ 1140	13
March/05/2013	human		feces	-	-	-
April/09/2013	laying hen group I	77	p.f.	+	HDZ 1141	52
	laying hen group II	94	p.f.	+	HDZ 1146	13
	laying hen group III	48	p.f.	+	HDZ 1151	13
	laying hen group IV	30	p.f.	-	-	-
	environmental sample	-	stored manure	+	HDZ 1153	13
December/18/2013	laying hen group I	27	p.f.	-	-	-
	laying hen group II	51	p.f.	+	HDZ 1180	13
	laying hen group III	84	p.f.	+	HDZ 1169	13
	laying hen group III	84	p.f.	+	HDZ 1178	13
	laying hen group III	84	p.f.	+	HDZ 1168	53
	laying hen group III	84	p.f.	+	HDZ 1179	53
	laying hen group IV	65	p.f.	+	HDZ 1172	53
	laying hen group IV	65	p.f.	+	HDZ 1199	53
	laying hen group IV	65	p.f.	+	HDZ 1204	55
	environmental sample of laying hen group I and II	-	p.d.	+	HDZ 1194	54
	environmental sample of laying hen of group III and IV	-	p.d.	-	-	-
	environmental sample	-	stored manure	+	HDZ 1184	13
February/10/2014	laying hen group I	35	p.f.	+	HDZ 1210	53
	laying hen group II	58	p.f.	-	-	-
	laying hen group III	20	p.f.	-	-	-
	laying hen group IV	73	p.f.	+	HDZ 1212	53
	laying hen group IV	73	p.f.	+	HDZ 1223	53
April/01/2014	laying hen group I	42	p.f.	+	HDZ 1224	13
	laying hen group I	42	p.f.	+	HDZ 1226	13
	laying hen group I	42	p.f.	+	HDZ 1225	53
	laying hen group II	66	p.f.	+	HDZ 1235	13
	laying hen group II	66	p.f.	+	HDZ 1233	53
	laying hen group II	66	p.f.	+	HDZ 1250	54
	laying hen group II	66	p.f.	+	HDZ 1228	56
	laying hen group II	66	p.f.	+	HDZ 1229	57
	laying hen group II	66	p.f.	+	HDZ 1230	57
	laying hen group III	28	p.f.	-	-	-
	laying hen group IV	80	p.f.	+	HDZ 1246	13
	laying hen group IV	80	p.f.	+	HDZ 1242	53
	laying hen group IV	80	p.f.	+	HDZ 1240	54
	laying hen group IV	80	p.f.	+	HDZ 1237	55
	laying hen group IV	80	p.f.	+	HDZ 1238	55
	laying hen group IV	80	p.f.	+	HDZ 1244	55

* +: positive cultivation on selective medium;-: negative cultivation on selective medium [25];

^(#)^: cultivation of the blood culture; see material and method section

** sequence type

p.f. pooled fecal samples, p.d. pooled dust samples

In the described study, only fecal droppings from the manure belt were used. The owner of the laying hens gave permission to use the fecal droppings of his laying hens. No specific permits were required for the described study. No purpose killing of laying hens or invasive experiments were performed in this study. No ethical approval for the analysis of animal samples was necessary.

#### Isolation from droppings, dust samples and stored manure

Pooled samples of five fresh droppings were sampled from the manure belts of each laying hen group. The droppings were transferred into a sterile stomacher bag and mixed well by kneading the contents. An amount of 2 g of feces from each sample was used for the cultivation of *S*. *gallolyticus* subsp. *gallolyticus*, as described previously [25]. Furthermore, two pooled dust samples were randomly taken from different locations in the laying hen flock (maximum distance to laying hens: 1 meter), as described previously [26]. A quantity of 0.1 g of each mixed dust sample was resuspended in 2 ml Dulbecco’s Phosphate-Buffered Saline (DPBS; Gibco Life Technologies, Karlsruhe, Germany). Two samples were taken from the stored manure and 2 g was used for the cultivation of *S*. *gallolyticus* subsp. *gallolyticus*. Both sample types were treated in the same way [25].

#### Isolation from human blood

The human blood was cultured by using a BacT/Alert3D continuous monitoring system (bioMérieux, Nürtingen, Germany). Aerobic and standard anaerobic culture bottles (bioMérieux, Germany) were inoculated with a 5 ml sample and were incubated at 37°C until a positive signal was detected. The bacterial isolate was cultured on Columbia agar with 5% sheep blood (bioMérieux, Germany).

#### Identification

Thirty-three suspected *S*. *gallolyticus* subsp. *gallolyticus* isolates (29 from laying hen feces, 1 from human, 3 from the environment (1 from dust, and 2 from stored manure)) were grown on brain heart infusion (BHI) agar at 37°C (Oxoid Ltd., Cambridge, UK) and subsequently characterized by MALDI-TOF MS and partial sequencing of the manganese-dependent superoxide dismutase gene (*sodA*) [23, 24].

### DNA extraction

The total DNA of *S*. *gallolyticus* subsp. *gallolyticus* strains was isolated with the QIAamp Blood Mini Kit (Qiagen, Hilden, Germany). The DNA extraction was performed in accordance with the manufacturer’s instructions.

### Multilocus sequence typing

Gene fragments of seven housekeeping genes of 33 *S*. *gallolyticus* subsp. *gallolyticus* isolates were amplified from chromosomal DNA. Primer systems for the following genes: *aroE*, *glgB*, *nifS*, *p20*, *tkt*, *trpD*, and *uvrA* were used [20].

PCR and sequencing were carried out, as described previously, and can be seen on the website www.pubmlst.org [20, 27]. Sequences were analyzed using BioNumerics software 6.6 (Applied Math, St-Martens-Latem, Belgium) and a UPGMA (unweighted pair group method with arithmetic mean) dendrogram was constructed to investigate the population structure. Based on the UPGMA dendrogram, a minimum spanning tree (MST) was created [20] and the allelic profiles were used to determine the Simpson’s index of diversity (SID; http://darwin.phyloviz.net/ComparingPartitions), the index of association (I_A_; START version 2 [28]) and clonal lineages (eBURST version 3; www.mlst.net; [29]) within the population.

### DNA fingerprinting

To identify clonal identity of *S*. *gallolyticus* subsp. *gallolyticus* DNA fingerprinting was performed using the ERIC2 primer [30] to classify 33 *S*. *gallolyticus* subsp. *gallolyticus* isolates. The detection of PCR products was performed by chip gel electrophoresis (Agilent 2100 Bioanalyzer (Agilent Technologies, Waldbronn, Germany)). The data of the chip gel electrophoresis were analyzed with the software DiversiLab (BioMérieux, Germany).

### Antibiotic resistance testing

Antibiotic resistance testing was performed according to standard procedures. In brief, a volume of 2 ml 0.9% sodium chloride (Braun, Melsungen, Germany) was inoculated with *S*. *gallolyticus* subsp. *gallolyticus* to an optical density of 0.5 McFarland from which 200 μl with 250 μl horse blood (Thermo Scientific, Wilmington, USA) were added to 11 ml Mueller-Hinton Boullion II (Merck, Eppelheim, Germany). The suspension was splitted á 100 μl per well of a commercially manufactured 96 well plate (Micronaut-S; Merlin Diagnostics, Bornheim-Hersel, Germany). It was incubated 1 to 2 days at 37°C and 5% CO_2_ and Minimal Inhibitory Concentrations (MIC) were determined.

The breakpoints of concentrations of antibiotics were determined according to the guidelines of EUCAST for *Streptococcus* Lancefield group A, B, C and G, the *Streptococcus viridans* group and *Enterococcus* spp. (because to date, there are no EUCAST guidelines for *S*. *gallolyticus* subsp. *gallolyticus*).

## Results

*S*. *gallolyticus* subsp. *gallolyticus* was identified as the causative agent by analyzing the resulting isolates from the positive blood culture of the infective endocarditis patient using MALDI-TOF MS and *sodA* sequencing [23, 24]. Taken patient’s medical history revealed that the man is a farmer and cares for four groups of approximately 1,200 laying hens in a multiage small colony-keeping system. The here presented data refer to a flock of laying hens which is regularly diagnosed by a veterinarian as healthy flock.

In order to identify the potential transmission pathways of *S*. *gallolyticus* subsp. *gallolyticus*, the laying hen flock was sampled over a twelve-month period (April 2013, December 2013, February 2014, and April 2014; Table 1, Fig 1). Selected isolates were identified as *S*. *gallolyticus* subsp. *gallolyticus* according to the same methods that were used previously. This study made use of the website http://pubmlst.org developed by K. Jolley [27]. Data for the MLST scheme are available at www.pubmlst.org.

Pullets were placed into the building at 18 to 22 weeks old. Prior stocking rows (1 out of 4) stayed empty until the new pullets were introduced into the multiage system. Each time new hens arrived at the farm, droppings from the new pullets were tested negative for *S*. *gallolyticus* subsp. *gallolyticus*. However, the bacterium was detectable in droppings from these laying hens after a few months by selective cultivation (Table 1). At the age of 27 weeks (December 2013), for example, fecal droppings of laying hen group I from breeder 2 first tested negative for *S*. *gallolyticus* subsp. *gallolyticus*. At the age of 35 weeks, (February 2014) the fecal samples of the same laying hens tested positive. In February 2014, fecal droppings were collected before the pullets were introduced into the system (laying hen group III) and droppings remained negative up to the age of 27 weeks (April 2014) (Table 1).

**Fig 2:**
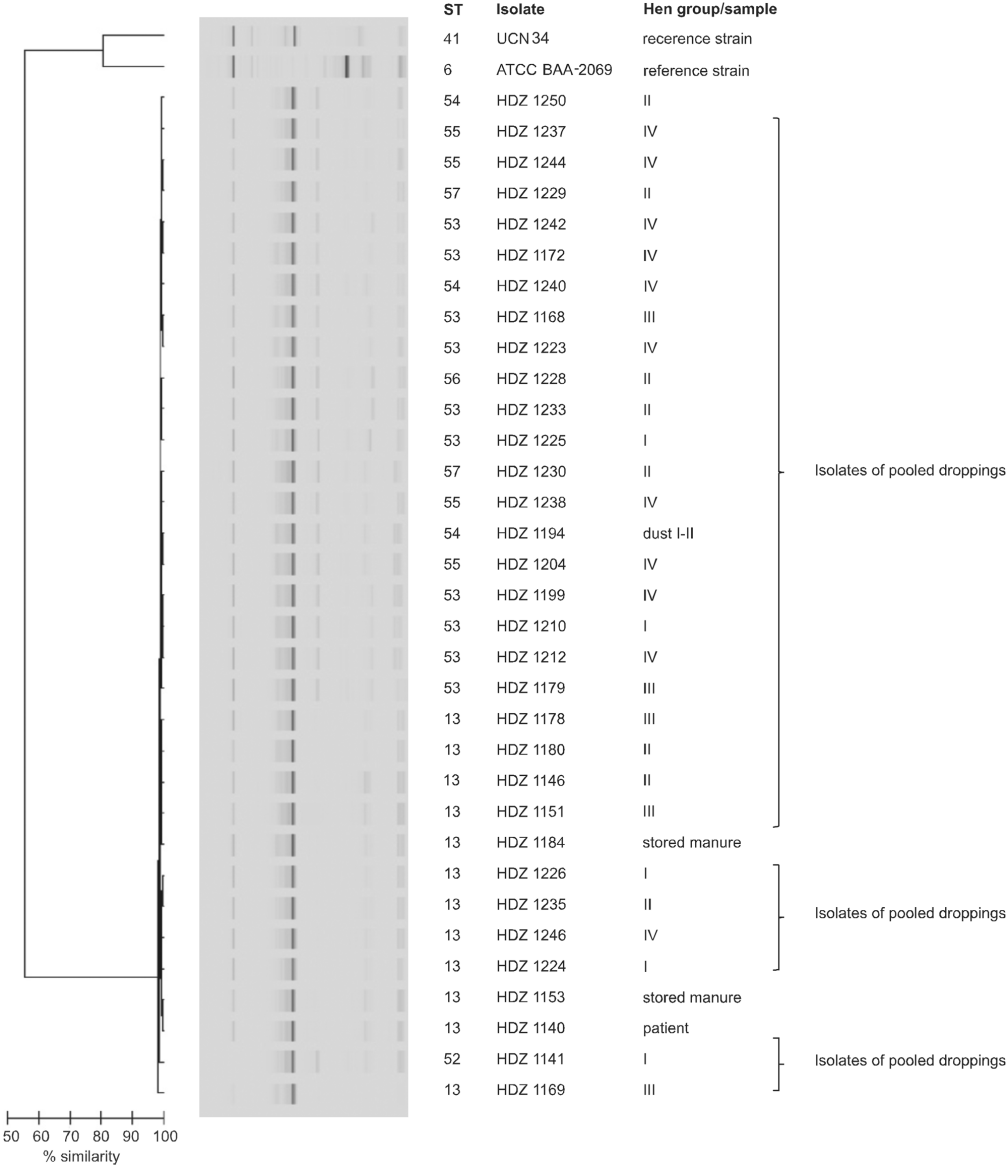
Dendrogram analysis and virtual gel images of DNA fingerprinting of *S*. *gallolyticus* subsp. *Gallolyticus*. 33 *S*. *gallolyticus* subsp. *gallolyticus* were analyzed by DNA fingerprinting. Chip gel electrophoresis (Agilent 2100 Bioanalyzer (Agilent Technologies, Waldbronn, Germany)) was used to detect PCR products. *S*. *gallolyticus* subsp. *gallolyticus* ATCC BAA-2069 [31] and UCN 34 [32] were used as reference strains to root the phylogenetic tree.

The human blood culture isolate, the isolates from the fecal droppings and from the environment were analyzed using MLST [20, 27] (Fig 1) and DNA fingerprinting (Fig 2).

Typing analyses of the seven housekeeping genes divided 33 isolates into seven STs (13, 52, 53, 54, 55, 56, and 57) that formed two distinct clusters (Fig 1, Table 1). The cluster border was defined based on the calculated average SID to get better insights into the population (Fig 1) [20].

ST 13 was found simultaneously in the human blood culture of the infective endocarditis patient, in the isolates of all laying hen groups with different ages and in the excrement of the stored manure at different time points (Fig 1, Table 1). Similar observations could be seen for ST 53, which was also found within all laying hen groups. In comparison to all other sequence types present, ST 13 could be detected over the entire period of sampling. ST 52 was only present in April 2013 (Fig 1, Table 1).

The UPGMA dendrogram showed the presence of isolates of the two different breeders within both defined clusters. *S*. *gallolyticus* subsp. *gallolyticus* isolates of hen groups originating from the same breeder had various sequence types. Breeder 1, especially, can be assigned to each sequence type (Fig 1).

Within these investigations *S*. *gallolyticus* subsp. *gallolyticus* had been isolated from particles of dust. The ST 54 isolate from dust was also identified within laying hen group II and IV (Fig 1, Table 1).

**Fig 3:**
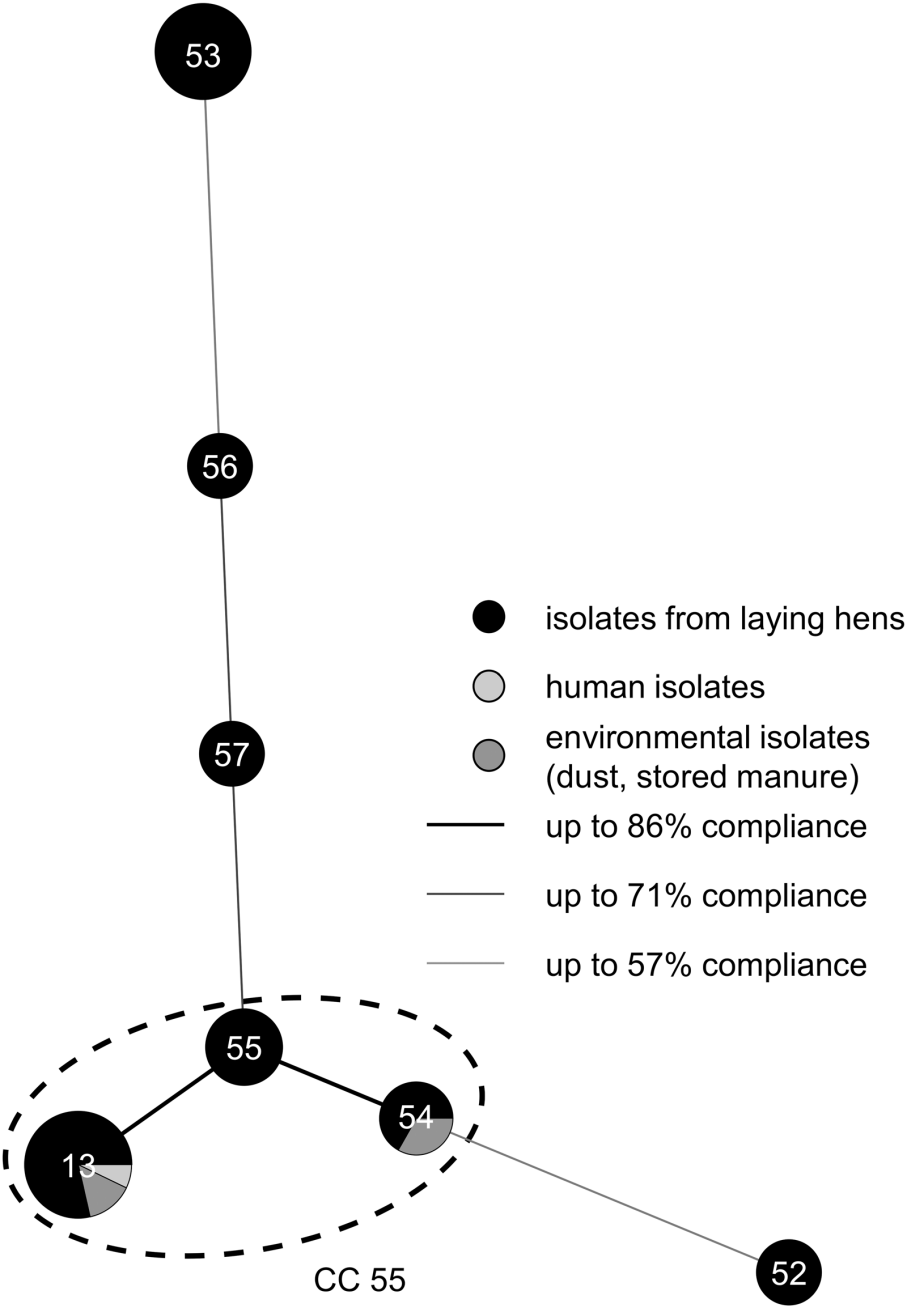
Clustering of 33 *S*. *gallolyticus* subsp. *gallolyticus* isolates by use of MST and eBURST. An MST was generated based on the UPGMA dendrogram. Each circle represents one ST. The size of a circle corresponds to the number of isolates included. The shadings indicate the origin of the *S*. *gallolyticus* subsp. *gallolyticus* isolates. The clonal complex (CC) was determined by eBURST and is symbolized by a dashed line (CC 55).

An MST was constructed based on the STs to characterize the phylogenetic relatedness of the *S*. *gallolyticus* subsp. *gallolyticus* isolates of the laying hen flock (Fig 3) [20]. ST 55 represents the origin of the population and was proposed as a predicted primary founder of the *S*. *gallolyticus* subsp. *gallolyticus* isolates of ST 13 and 54 using eBURST (Fig 3). These three STs are grouped into one clonal complex (CC 55). The remaining four STs are singletons. Determination of the SID (range: 0–1) resulted in a value of approximately 0.5 (95% confidence interval (CI), 0.310 to 0.716). A value close to 0 shows a low rate of diversity within the population characterized. The results were confirmed by using DNA fingerprinting analysis, which presented a high diversity between the reference strains ATCC BAA-2069 [31], UCN 34 [32] and the isolates of the flock. All strains, independent of the group, showed very similar DNA fingerprinting patterns (Fig 2). Furthermore, an index of association was calculated which resulted in a significant linkage disequilibrium.

In general, the isolates of laying hens as well as the human isolate have the same antibiogram. The isolates are sensitive to ampicillin, amoxicillin/clavulanic acid, ceftriaxone, cefuroxime, clarithromycin, doxycycline, erythromycin, imipenem, moxifloxacin and penicillin G. Three isolates are intermediary (HDZ 1141, HDZ 1210 and HDZ 1235) and all other isolates are resistant to ciprofloxacin.

In summary, seven STs were detected within the twelve-month period of sampling within the laying hen flock. Notably, newly introduced pullets always tested negative for *S*. *gallolyticus* subsp. *gallolyticus* and became positive with increasing age (Table 1). The STs 13, 53 and 54 occur in all groups of animals in the multiage laying hen flock. The 54^th^ ST was further cultivable from particles of dust and ST 13 from excrement of the stored manure (Fig 1, Table 1). Above all, the opportunistic organism with ST 13 was not only detected in the droppings of hens and in the excrement of the stored manure, but also in the blood culture of the animal caretaker with infective endocarditis (Fig 1, Table 1). Finally, the 33 isolates characterized here all presented a similar DNA fingerprinting pattern (Fig 2) and almost the same antibiogram.

## Discussion

*S*. *gallolyticus* subsp. *gallolyticus* was identified in a human blood culture of an animal caretaker who suffered from infective endocarditis. Furthermore, *S*. *gallolyticus* subsp. *gallolyticus* with the same allelic profile was detected in droppings of laying hens of his flock and in samples from the laying hen environment (dust, stored manure).

The fact that chickens may carry *S*. *gallolyticus* subsp. *gallolyticus* was already known [6, 21]. Thus, a potential transmission of *S*. *gallolyticus* subsp. *gallolyticus* between laying hens and the farmer may have occurred. However, it is still unknown how the bacterium can be transmitted. Consequently, subspecies-specific MLST, DNA fingerprinting and eBURST analyses were utilized for 33 isolates and indicate the first case of a potential *S*. *gallolyticus* subsp. *gallolyticus* transmission between laying hens and humans. Since the development of the MLST this method becomes the gold standard to clarify epidemiologic relationships of bacterial isolates. It is an equivalent method to the pulsed-field gel electrophoresis and can be used to investigate population structures [33, 34]. In addition, the DNA fingerprinting (ERIC PCR) was applied as complementary molecular genetic method to characterize the very similar STs in terms of the same origin of *S*. *gallolyticus* subsp. *gallolyticus* isolates.

In previous studies the bacterium was detected in the gut of other farm animals such as bovines, horses, pigs and dogs [5]. However, in this study, there is no mention of any other animals on the farm with the exception of an ST13 isolate also isolated from mouse droppings, which is discussed later. This is due to the fact, that this farm inhabits only laying hens and no other animals, preventing the investigation of other inter-farm animal and pet species transmission.

It was noticeable that each time new pullets were introduced into the flock, *S*. *gallolyticus* subsp. *gallolyticus* could not be detected within the new groups. In the case of *Campylobacter*, for example, a lag phase from two to three weeks was observed after the placement of chickens into a broiler house [35]. Based on the current data, it can be assumed that the *S*. *gallolyticus* subsp. *gallolyticus* colonization time of newly placed chickens may be longer. In laying hen group I, for example, *S*. *gallolyticus* subsp. *gallolyticus* was detectable after 35 weeks (Table 1). Therefore, *S*. *gallolyticus* subsp. *gallolyticus* may be transmitted from older hens to new hens, however, the precise transmission pathways remain unclear.

Hypotheses were made to identify potential pathways of the transmission of the bacterium [20]. In this study, the breeder, the responding date of delivery (season) and the laying hen flock were included into the analyses (Fig 1, Table 1).

If there is a link between the detection of the bacterium and the breeder or the date of delivery, the same ST would be expected for all the *S*. *gallolyticus* subsp. *gallolyticus* isolates. Due to the identification of various STs, there are no hints towards a dependence of transmission of the facultative pathogen from the breeder to the flock. The same can be determined for the date of delivery. Therefore, no correlation between the ST and the date of delivery was identified (Fig 1). These observations may be explained by two options. Firstly, birds from the different breeders carried *S*. *gallolyticus* subsp. *gallolyticus* but we did not detect the bacterium because of the low colonization rates of the laying hens. Secondly, birds were colonized within the farm by environmental colonization or the farmer transferred the bacterium from one group to another. This theory will be supported by the distribution of ST 13 and 53 within the laying hen flock, whereby it seems that these types are most common.

However, in order to substantiate these results, more detailed investigations (e.g. by cloacal swabs from all birds) are necessary in the future to ensure the transmission routes of *S*. *gallolyticus* subsp. *gallolyticus*. Although potential transmissions of, for example, *Campylobacter jejuni* from breeder hens to broiler chickens were described in the literature [36], it has not yet been confirmed for *S*. *gallolyticus* subsp. *gallolyticus*. Furthermore, it seems to be that there is no link between the *S*. *gallolyticus* subsp. *gallolyticus* detection and the season. STs of isolates can be identified independently of the age of laying hens. ST 13 and ST 53, for example, are associated with the following dates: April and December 2013 and April 2014 (Fig 1, Table 1).

If the detection of *S*. *gallolyticus* subsp. *gallolyticus* may be independent of the breeders and the season, the presence of *S*. *gallolyticus* subsp. *gallolyticus* in the flock may be expected. The environment and the animal caretaker may be a possibility of a transmission between the laying hens, humans and the surroundings of the flock. A close relationship between the infected hens and the environment was demonstrated for *Salmonella* with the same phage type, thus, the environment seems to function as a reservoir for bacteria [26]. It is known that 2 to 8% of airborne dust particles in laying hen houses originate from excrement [37]. To date, there is no literature on the isolation of *S*. *gallolyticus* subsp. *gallolyticus* from environmental samples. It must be mentioned that this was the first time, to the best of our knowledge, that *S*. *gallolyticus* subsp. *gallolyticus* was isolated from particles of dust. This highlights the possibility of survival outside the gastrointestinal tract, which may contribute to an indirect transmission of *S*. *gallolyticus* subsp. *gallolyticus* from the surroundings to new pullets. This may be the reason for the detection of the same ST from fecal droppings of birds from different groups and different ages. It was shown that an effective cleaning in multiage systems is difficult to perform [38, 39]. Consequently, the risk of colonization of new pullets can be higher in these systems compared to all-in, and all-out systems. In this case, the transmission of contaminated dust from an occupied area to an already cleaned area for the new hens was possible. The fact that infectious agents may survive in empty and cleaned poultry houses was described, for example, for *Salmonella enteritidis* or *S*. *typhimurium* [40, 41]. Another source of colonization of laying hens in the flock may be the transmission of the opportunistic bacterium by living vectors, such as rodents. These vectors are known to contribute to *Salmonella* or *Campylobacter* infections in laying hen houses [26, 35, 42]. Thereby, it is assumed that birds become infected after ingesting feed contaminated with rodent feces [42]. Within this study, we further observed that mice may be colonized with *S*. *gallolyticus* subsp. *gallolyticus*. Fecal samples of mice were found onto the manure belt. Even it was not the focus of our study, isolates of pooled mice droppings from the laying hen flock were typed as ST 13. In this regard, a secondary contamination of droppings of mice, for example, by dust, cannot be excluded and, therefore, investigating the role of rodents as carriers of *S*. *gallolyticus* subsp. *gallolyticus* is recommended in the future.

In addition to these mechanisms, an oral fecal transfer may be considered. The manure of the small colony-keeping system is removed mechanically by a manure belt below the aviaries. Hence, birds may acquire colonization by direct contact with the excrements of other laying hens.

Working on a laying hen farm leads to contamination of the animal caretaker’s clothes directly by feces particles or indirectly by dust. Therefore, the distribution of the bacterial isolates may be supported by inadequate staff compliance (including not changing outer clothes, not disinfecting boots, not wearing gloves or masks (not mandatory)) or hygiene management (e.g. usage of the same utensils) [35] and clothes are not changed when leaving the hen house. Consequently, the bacterium can be potentially carried into the private household or between the laying hen groups via clothing or utensils. Such a link was shown for *Campylobacter* spp. in broiler chickens and laying hens in tropical climates with low-biosecurity housing [43].

Moreover, transmission rates of the bacterium between animals and humans might be increased through closer contact to colonized animals with *S*. *gallolyticus* subsp. *gallolyticus*. These relations were shown, for example, for livestock-associated *Staphylococcus aureus*, which can overcome species barriers and can be transmitted from animals to humans with direct livestock exposure [44, 45]. In this case it is possible that *S*. *gallolyticus* subsp. *gallolyticus* may be transmitted between different species, and it seems reasonable to suppose that *S*. *gallolyticus* subsp. *gallolyticus* increases the likelihood of colonizing or infecting laying hens or humans. But, it must be noted, that the colonization of laying hens with *S*. *gallolyticus* subsp. *gallolyticus* cannot be equated with an infection.

In addition to these observations, phylogenetic relationships of *S*. *gallolyticus* subsp. *gallolyticus* were studied using an eBURST algorithm. This algorithm is a model to give information about clonal complexes: a given genotype frequently increases in the population because of fitness advantages or genetic drift, resulting in the founder clone of the population [29,46]. This genotype can diversify by mutation and recombination and establishes the clonal complex of phylogenetic-related bacteria [29,46]. eBURST proposes ST 55 as the predicted primary founder, which might be explained through a fitness advantage (e.g. survival on dry surfaces). Consequently, these isolates increase in the population and become the founders of the clonal complex. The spread of these *S*. *gallolyticus* subsp. *gallolyticus* isolates (ST 13, ST 54) may be the result of biological fitness in contrast to other isolates from the droppings of laying hens. This might be an explanation for the continuous occurrence of isolates with ST 13 over the twelve-month period and the transmission between hens and animal caretaker.

In addition, a similar population structure was identified by DNA fingerprinting, which was confirmed by a low value of the SID (low rate of diversity) (Fig 2). The index of association calculated demonstrates low levels of horizontal gene transfer and mutations within the *S*. *gallolyticus* subsp. *gallolyticus* population of the laying hen farm. These data reflects a high phylogenetic relationship of all isolates. Another important indicator supporting the clonal identity of the isolates is the identical antibiotic resistance profile.

**Fig 4:**
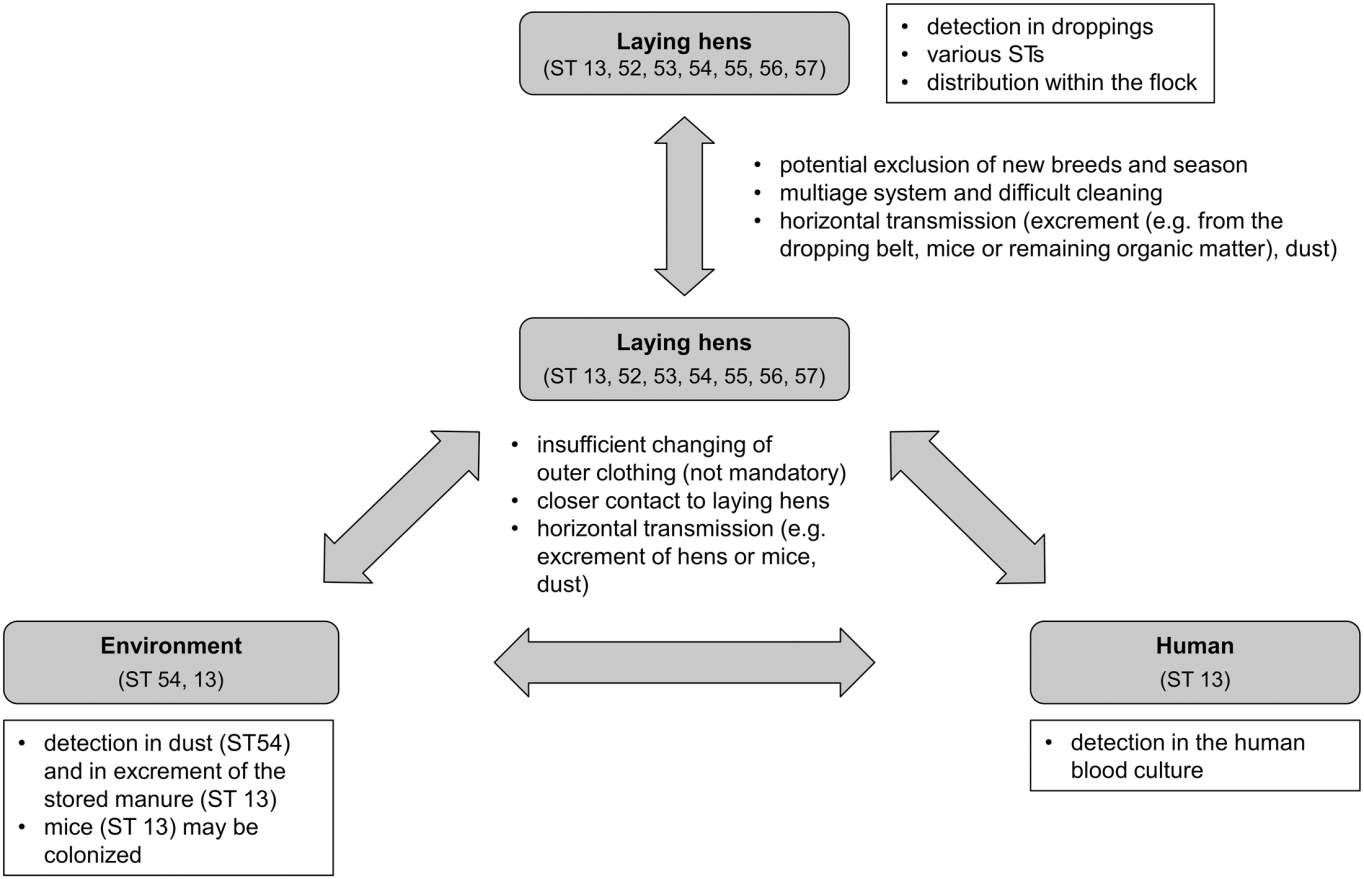
Potential transmission pathways of *S*. *gallolyticus* subsp. *gallolyticus* in a laying hen flock. General results of the identification of *S*. *gallolyticus* subsp. *gallolyticus* can be seen in the black framed white boxes. The transmission (symbolized by grey arrows) of the bacterium between laying hens themselves, between laying hens and humans, or between laying hens/humans and the environment may be supported by several factors, which are indicated as key points next to the arrows.

In conclusion, the systematic approach resulted in a more representative view concerning the direct and indirect transmission routes and the zoonotic potential of *S*. *gallolyticus* subsp. *gallolyticus*. Fig 4 shows a model that summarizes the findings of these investigations and of the potential transmission routes of *S*. *gallolyticus* subsp. *gallolyticus*. The latter was identified using the established subspecies-specific MLST. We reported, for the first time, the potential transmission of *S*. *gallolyticus* subsp. *gallolyticus* between laying hens and humans. The ST of the human blood culture isolate of the infective endocarditis patient was further identified in his laying hen flock. Thereby, the facultative pathogen may also be transmitted between laying hens and between laying hens/humans and the environment. Several factors may influence the transfer of the bacterium. The multiage system may support the colonization of new hens because of the problematic cleaning between the removal of one laying hen group and placement of new birds. *S*. *gallolyticus* subsp. *gallolyticus* can potentially horizontally transmit oral fecal by the excrement of laying hens, by the droppings of mice or by dust particles. The indirect transmission routes of the bacterium may be further influenced due to the animal caretaker of the flock, for example, by insufficient changes of outer clothing (not mandatory) and by the closer contact to laying hens. The typing of bacterial isolates is an epidemiological tool to describe the occurrence and transmission of potential zoonotic pathogens. This study showed the same ST of isolates of an infected person who had close contact with his colonized laying hens. Although, this is no confirmation of a transmission between the hens and the animal caretaker, a hint of a transfer was shown. However, further investigations seem to be necessary to assess the risk of a transmission of *S*. *gallolyticus* subsp. *gallolyticus* between animals and humans. Above all, future investigations of inter-farm animal and pet species transmissions are further interesting aspects to identify transmission routs of the facultative pathogen.
